# The prevalence, incidence and risk factors of herpes simplex virus type 2 infection among pregnant Zimbabwean women followed up nine months after childbirth

**DOI:** 10.1186/1472-6874-10-2

**Published:** 2010-01-12

**Authors:** Marshall W Munjoma, Edith N Kurewa, Munyaradzi P Mapingure, Grace V Mashavave, Mike Z Chirenje, Simbarashe Rusakaniko, Akhtar Hussain, Babill Stray-Pedersen

**Affiliations:** 1 College of Health Sciences, University of Zimbabwe, Department of Obstetrics and Gynaecology, Harare, Zimbabwe; 2 University of Oslo and Division of Obstetrics and Gynecology, Rikshospitalet, University Hospital, Norway; 3 College of Health Sciences, University of Zimbabwe, Department of Community Medicine, Harare, Zimbabwe; 4 Department of International Health, Institute of General Practice and Community Medicine, University of Oslo, Norway

## Abstract

**Background:**

Herpes simplex virus type 2 (HSV-2) is the leading cause of genital ulcer disease worldwide. The virus can be transmitted to neonates and there are scarce data regarding incidence of HSV-2 among women in pregnancy and after childbirth. The aim of this study is to measure the incidence and risk factors for HSV-2 infection in women followed for 9 months after childbirth.

**Methods:**

Pregnant women were consecutively enrolled late in pregnancy and followed at six weeks, four and nine months after childbirth. Stored samples were tested for HSV-2 at baseline and again at nine months after childbirth and HSV-2 seropositive samples at nine months after childbirth (seroconverters) were tested retrospectively to identify the seroconversion point.

**Results:**

One hundred and seventy-three (50.9%) of the 340 consecutively enrolled pregnant women were HSV-2 seronegative at baseline. HSV-2 incidence rate during the 10 months follow up was 9.7 (95% CI 5.4-14.4)/100 and 18.8 (95% CI 13.9-26.1)/100 person years at risk (PYAR) at four months and nine months after childbirth respectively. Analysis restricted to women reporting sexual activity yielded higher incidence rates. The prevalence of HSV-2 amongst the HIV-1 seropositive was 89.3%. Risk factors associated with HSV-2 seropositivity were having other sexual partners in past 12 months (Prevalence Risk Ratio (PRR) 1.8 (95% CI 1.4-2.4) and presence of *Trichomonas vaginalis *(PRR 1.7 95% CI 1.4-2.1). Polygamy (Incidence Rate Ratio (IRR) 4.4, 95% CI 1.9-10.6) and young age at sexual debut (IRR 3.6, 95% CI 1.6-8.3) were associated with primary HSV-2 infection during the 10 months follow up.

**Conclusions:**

Incidence of HSV-2 after childbirth is high and the period between late pregnancy and six weeks after childbirth needs to be targeted for prevention of primary HSV-2 infection to avert possible neonatal infections.

## Background

Herpes Simplex Virus type 2 (HSV-2) infection is a sexually transmitted infection (STI) which is recognized as the most common cause of genital ulcer disease worldwide [1,2]. Many people that are infected with HSV-2 are unaware of their infectious status in spite of symptoms [3]. The risk of acquiring HIV is greater with recent HSV-2 infections than with chronic infections [4]. HSV-2 prevalence, which is high in sub-Saharan Africa, occurs more frequently in women than in men [5] and is mainly transmitted through heterosexual contact. In the USA and in Norway about 2% and 2.6% respectively of susceptible women acquired HSV infection during pregnancy and those that acquire the infection close to term are at high risk of transmitting the virus from cervix or lower genital tract to their babies during vaginal delivery with the most serious consequences for the neonates [6,7]. Transplacental passage of virus is however rare and thus HSV-2 infection is not associated with stillbirths [8].

Earlier studies in Zimbabwe reported an HSV-2 prevalence of 42.2% amongst women of childbearing age [9], prevalence and incidence rates of 39.8% and 6.2/100 PYAR respectively amongst male factory workers [10]. The major public health importance of HSV-2 relates to its potential role in enhancing HIV transmission. The population attributable risk for HIV-1 due to HSV-2 in Zimbabwe is estimated at 65% [11] and for that reason HSV-2 infection should be recognized as a much greater public health problem than is currently the case. There are currently no studies in the sub-Saharan Africa to measure HSV-2 incidence rates and risk factors amongst women who have recently given birth. The postpartum period is a time when women are potentially more susceptible to STIs due to the traumatic nature of the vaginal delivery [12] and subsequent lack of oestrogen during lactation. Furthermore there may be unprotected sex among couples that ignore the dual protection against pregnancy and STIs offered by condoms and use them for contraceptive purposes only since part of this period is often considered "safe" from falling pregnant. The purpose of this study is to measure the incidence rate and prevalence of HSV-2 among women followed 9 months after childbirth.

## Methods

Between April and September 2002, 354 consecutive pregnant women seeking routine antenatal services from three randomly selected primary health care clinics in two of Harare's peri-urban high density suburbs were invited to participate in the study on average four weeks before childbirth. Five (5) of the women refused to participate, six (6) agreed but did not turn up for any of the scheduled visits and three (3) only turned up at the six week visit but no samples were taken. This analysis was based on the 340 women (173 HSV-2 uninfected and 167 HSV-2 infected) that came at all the scheduled visits.

The women were enrolled from the national program for the prevention of mother to child transmission of HIV at around 36 weeks of gestation and were followed up to investigate the role of STIs and micronutrients on mother to child transmission of HIV. The women were enrolled if they were pregnant, willing to undergo HIV counseling and testing, had no history of complications with the current pregnancy and were planning to deliver at any of the three randomly selected clinics. HSV-2 was tested at baseline and samples that were HSV-2 seronegative were tested for HSV-2 seroconversion at nine months after childbirth. Samples that were seropositive at nine months were tested retrospectively at four months and six weeks to identify the last seronegative and first seropositive visit. The study participants were encouraged to bring their male partners for counseling, testing and treatment of curable STIs. All the participants consented to long term storage and future testing of their samples. All participants provided written informed consent and the study was approved by both the Medical Research Council of Zimbabwe and the Norwegian Ethical Committee.

At baseline female interviewers administered a standardized questionnaire to record socio-demographic, reproductive history and sexual behavior information. A gynaecological examination was done by a trained clinician during which a high vaginal swab for testing of *Trichomonas vaginalis *and yeast cells was collected together with a blood sample for testing HSV-2, HIV-1 and syphilis. A structured questionnaire was administered at follow up visits mainly to capture information relating to the period since the last visit.

Antibodies to HSV-2 at baseline were identified using the HerpeSelect 2 ELISA IgG (Focus Diagnostics) and the results were interpreted according to the manufacturers' specifications while all the follow up tests were interpreted using an index cut-off value of 3.5. Antibodies to HIV-1 were identified using two rapid test kits in parallel, "Determine" (Abbott Diagnostics) and Oraquick (Orasure technologies). Screening for syphilis was done using a non-specific rapid plasma reagin (RPR) and all positives were confirmed using Treponema Pallidum Hemaglutination Assay (TPHA), both manufactured by Randox Laboratories. A wet mount prepared from freshly collected vaginal fluid was mixed with normal saline and examined using a compound microscope for the presence of *Trichomonas vaginalis *and yeast cells.

### Statistical analyses

Data were analyzed with the Statistical Package for Social Sciences (SPSS) version 16.0 (SPSS, IL, USA) and STATA version 10.0, Texas, USA. Incidence, as a percentage and expressed as person years at risk (PYAR) was calculated for everyone and restricted analysis was done on participants that reported having resumed sexually activity after childbirth. Time of infection was assumed to have occurred mid point between last negative and first positive test. Because the HSV-2 prevalence was high we calculated prevalence risk ratios (PRR) using log binomial regression with the generalized linear model (glm) function in STATA. Poisson regression and the robust option of estimating variance-covariance matrix was used to calculate incidence rate ratios (IRR) for factors associated with HSV-2 acquisition over the ten months follow up period. Multivariate regression with cut off set at p = 0.25 was performed in STATA to investigate independent predictors of HSV-2 seropositivity. Statistical significance was set at p < 0.05 and Fisher's exact p-values were used where appropriate.

## Results

Three hundred and forty (340) pregnant women agreed to participate and had samples collected during the 10 months follow-up period from enrolment to nine months after childbirth. The median age of the participants at enrollment was 23.0 years with an interquartile range of 20-28 years; median number of pregnancies (including current pregnancy) was 2.0 and median number of living children was 1.0. The majority of the women (97.9%) were married but of note were the 9% (95% CI 6.0-12.1) who were in polygamous marriages. Although the participants were encouraged to bring their male partners only 43 males (12.6%) attended.

The prevalence of HSV-2 and HIV-1 amongst participants at enrollment was 49.1% and 24.7% respectively whilst the prevalence of HSV-2 among the 43 male partners was 46%. The prevalence of syphilis as confirmed by TPHA was 0.3%. Table 1 shows the characteristics that are associated with prevalent HSV-2 infection. Being in a polygamous marriage (PRR 1.4, 95% CI 1.1-1.9), having other sexual partners in the past 12 months (PRR 1.8, 95% CI 1.4-2.4), having ever used contraceptives (PRR 1.5, 95% CI 1.2-2.0), being infected with *Trichomonas vaginalis *(PRR 1.7, 95% CI 1.4-2.1) and HIV-1 (PRR 2.5 95% CI 2.1-3.0) all have statistically significant association with HSV-2 prevalence in univariate analysis.

**Table 1:** Univariate analysis of risk factors for HSV-2 positivity among pregnant women

Variable	n	HSV-2 Positive	Prevalence Risk Ratio (PRR) (95% CI)	p value
Total	340	167(49.1%)		
Age group				
<20 yrs	65	25(38.5%)	Referent	
20-24 yrs	135	55(40.7%)	1.1(0.7-1.5)	0.760
25-29 yrs	76	47(61.8%)	1.6(1.1-2.3)	0.009
30-34 yrs	47	33(70.2%)	1.8(1.3-2.6)	0.001
>34 yrs	17	7(41.2%)	1.1(0.6-2.0)	0.836
Marital Status*				
Single	7	2(28.6%)	Referent	
Married	330	163(49.4%)	1.7(0.5-5.6)	0.362
Polygamy				
No	303	144(47.5%)	Referent	
Yes	31	21(67.7%)	1.4(1.1-1.9)	0.010
Educational level				
>Primary	279	131(47.0%)	Referent	
≤Primary	61	36(59.0%)	1.3(1.0-1.6)	0.066
Age at sexual debut*				
≥16	298	145(48.7%)	Referent	
<16	38	20(52.6%)	1.1(0.8-1.5)	0.634
Other sexual partners past 12 months				
No	329	157(47.7%)	Referent	
Yes	8	7(87.5%)	1.8(1.4-2.4)	< 0.001
Ever used contraceptives*				
No	136	51(37.5%)	Referent	
Yes	203	116(57.1%)	1.5(1.2-2.0)	0.001
Ever used intravaginal herbs*				
No	283	136(48.1%)	Referent	
Yes	56	31(55.4%)	1.2(0.9-1.5)	0.295
Clinical genital ulcer*				
No	282	131(46.5%)	Referent	
Yes	8	6(75.0%)	1.6(1.1-2.5)	0.025
Clinical genital warts*				
No	270	125(46.3%)	Referent	
Yes	25	15(60.0%)	1.3(0.9-1.8)	0.141
*Trichomonas vaginalis*				
Negative	296	134(45.3%)	Referent	
Positive	38	30(79.0%)	1.7(1.4-2.1)	< 0.001
Yeasts*				
Absent	204	102(50.0%)	Referent	
Present	130	62(47.7%)	1.0(0.8-1.2)	0.682
HIV-1 serostatus at enrolment				
Negative	256	92(35.9%)	Referent	
Positive	84	75(89.3%)	2.5(2.1-3.0)	< 0.001

*denominator less than 340.

In multivariate analysis HIV positivity and infection from *Trichomonas vaginalis *remained as independent significant risk factors for HSV-2 seropositivity with risk estimates of 2.4(1.9-3.0) and 1.7(1.3-2.2) respectively.

**Figure 1:**
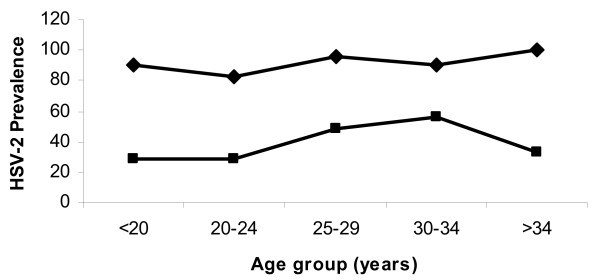
**Prevalence of HSV-2 amongst HIV infected and uninfected pregnant women according to age groups**.

Figure 1 shows distribution of HSV-2 seropositivity by age group among the HIV-1 infected (black diamond) and HIV uninfected (black square) participants. As observed the prevalence of HSV-2 among the HIV-1 infected was above 80% across all age groups. Of note is the relatively low HSV-2 prevalence among the HIV uninfected (35.0%) especially among those aged >34 years. The overall prevalence of HSV-2 amongst HIV-1 infected participants was 89.3% compared to 35.8% amongst the HIV-1 uninfected, PRR 2.5(2.1-2.9).

During the 10-month follow-up period, 20 of the 167 seronegative women acquired HSV-2, seven (7) was detected at four months and 13 at nine months after childbirth. At six weeks three (3) had an index value > than 1.1 but < 3.5 and were therefore considered not seroconverted at that visit but were confirmed and included in the four months visit. Table 2 shows the incidence rate of HSV-2 seroconversion per 100 PYAR for all seronegative mothers and for those that reported being sexually active at different times after childbirth. From four months to nine months after childbirth, sexually active mothers reported a higher incidence rate of 21.2/100 PYAR (table 2). The cumulative incidence rate during the 10 months was 13.9/100 PYAR. Nineteen (19) of these seroconverters (11.6%) were among the 164 HIV-1 negative women while one seroconverter occurred among the 9 (11.1%) HIV-1 positive women, p = 0.955. Most of the seroconverters (80%) were below 25 years of age.

**Table 2:** HSV-2 seroconversion at different time points after childbirth

Period observed	Enrollment - 4 months pp* (5 months)	4-9 months pp* (5 months)	Enrollment - 9 mths pp* (10 months)
Seroconverters, n	7	13	20
Seronegative women, n	173	166	173
Person-time	72.1 person-years	69.2 person-years	144.2 person-years
Incidence per			
100 PYAR* (95%CI)	9.7 (5.4-14.3)	18.8 (13.9-26.1)	13.9 (9.1-19.6)
Sexually active mothers, n (%)	144 (83.3%)	147 (88.4%)	153 (88.4)%
Person-time	60.0 person-years	61.3 person-years	127.5 person-years
Incidence per 100 PYAR* in sexually active mothers (95%CI)	11.7 (6.5-16.9)	21.2 (16.2-294)	15.7 (10.9-22.8)

*PYAR - Person years at risk, *pp - postpartum

Table 3 shows the risk factors for acquisition of HSV-2 within the 10 months follow up and of note is the statistically significant risk of women in polygamous marriages compared to those in monogamous marriages (IRR 4.4 95% CI 1.9-10.6). Low age at sexual debut was significantly associated with acquisition of HSV-2 (IRR 3.6 95% CI (1.6-8.3). Over 95% of the women in each group cleansed the vagina with water. Mean age (SD) difference for non-seroconverters and their partners and seroconverters and their partners were 5.5 (4.7) and 8.7 (5.8) respectively, p = 0,008. IRR for seroconverting per unit increase in age difference between the women and their partners was 1.07 (95% CI 1.0 - 1.1).

**Table 3:** Univariate analysis of risk factors for seroconverting within 9 months after childbirth

Variable	n	HSV-2 seroconversion	Incidence Risk ratio (IRR) (95%CI)	P value
Total	173	20 (11.7)		
Age group				
<20 yrs	40	6(15.0%)	Referent	
20-24 yrs	81	10(12.4%)	0.8(0.3-12.1)	0.685
25-29 yrs	29	1(3.5%)	0.2(0.0-1.8)	0.164
30-34 yrs	13	2 (15.4%)	1.0(0.2-4.5)	0.973
>34 yrs	10	1(10.0%)	0.7(0.1-5.0)	0.692
Marital status*				
Single	5	0	Referent	
Married	167	20(12.0%)	Incalculable	1.000**
Polygamy				
No	160	16(10.0%)	Referent	
Yes	9	4(44.4%)	4.4(1.9-10.6)	0.001**
Educational level				
>Primary school	149	17(11.4%)	Referent	
= <Primary school	24	3(12.5%)	1.1(0.3-3.5)	0.877**
Age at sexual debut (years)*				
>16 years	153	14 (9.2%)	Referent	
≤16 years	18	6 (33.3%)	3.6(1.6-8.3)	0.002
Abstain from sex during pregnancy*				
Yes	40	4 (10.0%)	Referent	
No	131	16 (12.2%)	1.2 (0.4-3.5%)	0.706**
Resumed sex after delivery*				
Six weeks after birth				
No	88	11(12.6%)	Referent	
Yes	40	6 (15.0%)	1.2 (0.5-3.0)	0.699
Four months after birth				
No	10	1 (10.0%)	Referent	
Yes	74	10 (13.5%)	1.4 (0.2-9.6)	0.763**
Nine months after birth				
No	12	0 (0)	Referent	
Yes	131	12 (9.2)	Incalculable	0.273**
Cleanse the vagina*				
No	7	0 (0)	Referent	
Yes	166	20 (12.1)	Incalculable	0.329**
Ever used intravaginal herbs*				
No	147	16 (10.9%)	Referent	
Yes	25	4 (16.0%)	1.5 (0.5-4.0)	0.461**
Clinical genital ulcer*				
No	151	18 (11.3%)	Referent	
Yes	2	0	Incalculable	0.615**
Clinical genital warts*				
No	146	15 (10.4%)	Referent	
Yes	9	2 (22.2%)	2.2 (0.6-8.1)	0.251**

*denominator less than 173. ** Fisher's Exact p values.

HSV-2 infected women were on average significantly older than HSV-2 uninfected women, mean ages 25.5 and 22.9 years respectively, p < 0.001. Similarly male partners of HSV-2 infected women were on average significantly older than male partners of HSV-2 uninfected women, mean ages 31.9 and 28.4 years respectively, p < 0.001. Mean age difference for the partnership between HSV-2 infected and HSV-2 uninfected were 6.4 and 5.5 years respectively, p = 0.026.

## Discussion

Our results showed a cumulative HSV-2 incidence of 13.9/100 PYAR at 9 months after childbirth, which is higher than previously reported figures in literature [9,10]. If the risk of acquiring HIV-1 is greater amongst HSV-2 primary cases compared to chronic cases [4] then our data demonstrate that HSV-2/HIV-1 seronegative women that acquire HSV-2 postpartum have the highest risk of acquiring HIV-1. This high HSV-2 incidence may be due to a combination of two factors; firstly this group of women may be using condoms purely for contraceptive purposes and stopped during pregnancy and breastfeeding period and secondly, vaginal delivery is an event that leaves the birth canal traumatized [12] thereby making women more vulnerable to STIs if they have unprotected sex. The low syphilis prevalence observed could be due to the fact that syphilis screening is standard of care for pregnant women in Zimbabwe.

The risk factors associated with acquisition of HSV-2 after giving birth were polygamous marriage and young age at sexual debut. In Zimbabwe the hazard ratio for HIV among HSV-2 seroconverting women is 8.6 (95% CI 4.3-17.1) compared with 4.4 (95% CI 2.7-7.2) among women with chronic HSV-2 [11]. With a fertility rate of 3.8 children [13] a high HSV-2 incidence after birth implies that each time a woman gives birth she goes through a period when the risk of acquiring HSV-2 is very high. Furthermore, the risk of neonatal herpes is greatest in a pregnant woman who acquires HSV infection late in pregnancy [6,14] due to lack of circulating maternal antibodies that usually provide the fetus with passive immunity [15]. Our participants, particularly the three that were below an index value of 3.5 at six weeks, could have been infected late in pregnancy and thus were at risk of transmitting HSV-2 to their unborn infants. However there was no evidence of clinical neonatal herpes infection in these children.

There was no difference between rates of acquisition of HSV-2 amongst HIV-1 infected (11.1%) compared to HIV-1 unifected (11.7%). Several other studies have demonstrated the role of HSV-2 in the transmission of HIV-1 [11,16,17]. However, a recent study which tested stored samples collected in the early 80's showed that the prevalence of HSV-2 in this part of the world was already widespread before the HIV epidemic and has not been greatly affected by the HIV-1 epidemic [18]. People that are co-infected have more frequent HSV-2 reactivations which last longer [19] and tend to shed more herpes virus than HIV-1 uninfected [20].

The prevalence for HSV-2 and HIV-1 was 49.4% (44.1-54.7) and 24.9% (20.2-29.5) respectively. Contrasting prevalence of two infections that share essentially the same risk factors is a result of the combination of the infectiousness of HSV-2 and the high mortality associated with HIV-1 disease [21] in regions where availability of HIV/AIDS treatment is limited. HSV-2 targets the epithelial cells during the infectious process [22] while HIV-1 targets mainly CD_4_+ lymphocytes, which can mostly be reached via lesions in the mucosa. In 2007 about 67% of the 32.9 million people living with HIV-1 were in the sub-Sahara where 75% of all HIV related deaths occurred [21]. In Zimbabwe the population attributable fraction of HIV due to HSV-2 is about 65% [11] and as a result HSV-2 should be recognized as a public health problem leading to design and implementation of HSV-2 prevention strategies such as health education about the role of HSV-2 and how it enhances HIV infectiousness.

When stratified by age the prevalence of HSV-2 was seen to be high amongst the HIV-1 infected (black diamond) across all age groups compared to HIV-1 uninfected (black square) participants (Figure 1). Furthermore HSV-2 seropositive women were about three times more likely to be HIV-positive than HSV-2 negative women. The disparities in HSV-2 prevalence between the HIV-1 infected and uninfected indicate an increased susceptibility of the HSV-2 seropositive to HIV-1 infection [9,23]. Because of the bidirectional association between HIV and HSV-2 it follows that HIV infected/HSV-2 uninfected are also at risk of acquiring HSV-2 [10,24]. If these women acquire HSV-2 infection late in pregnancy their risk of transmitting the virus to the infant intrapartum is high [6,25]. In such cases, if the couple is HSV-2 discordant, we recommend that they avoid sexual contact late in pregnancy. If avoiding sexual contact late in pregnancy is difficult then we suggest prophylactic treatment with acyclovir to HIV infected/HSV-2 uninfected pregnant women to avoid neonatal herpes [26], especially if their partners are HSV-2 infected.

The majority of the participants had their sexual debut before the age of 20. Young women have the highest risk of acquiring STIs including HIV-1 infection [27]. Early sexual debut and polygamy were significantly associated with acquisition of HSV-2 after childbirth. The Southern African region is currently using the "OneLove" approach to discourage people from having multiple concurrent partners(MCP) [28]. Multiple concurrent partnerships are observed when older men have sexual relationships with young girls and vice versa. Polygamy should be considered as having MCP and should be discouraged.

Age plays an important role in the transmission of STIs. In this study we found that HSV-2 infected participants as well as their partners were significantly older than the HSV-2 uninfected participants and their partners. Age difference between women and their husbands remains a silent driver of the HIV-1 epidemic as the older men have more exposure than the young women, resulting in a disproportionate prevalence of STIs between young men and women [27,29,30].

Although the study participants were encouraged to bring their male partners for counseling, testing and treatment of curable STIs only 43 (12.6%) came. In sub-Saharan Africa reproductive health research place a disproportionate emphasis on women and ignore the equally important role of men [31]. Poor attendance by men in this study (12.8%) should encourage researchers to come up with strategies to involve men to participate in reproductive health research.

## Conclusions

Incidence of HSV-2 after childbirth is high and therefore the period between late pregnancy and six weeks after childbirth needs to be targeted for prevention of primary HSV-2 infection to avert possible neonatal infections. Ideally HSV-2-discordant couples must be identified and advised to avoid sexual contact late in pregnancy. Furthermore effort must be made to include men in reproductive health research.

## Competing interests

The authors declare that they have no competing interests.

## Authors' contributions

MM was involved in designing, concepting and drafting of the manuscript. EK assisted in concepting and drafting of the manuscript. PM and SR were involved in the analysis and interpretation of data. MC and AH were involved in concepting, analysis and interpretation of data. GM was involved in acquisition and interpretation of laboratory data while BS-P was involved in concepting, designing, analysis and drafting of manuscript. All the authors read and approved the final manuscript.

## Pre-publication history

The pre-publication history for this paper can be accessed here:

http://www.biomedcentral.com/1472-6874/10/2/prepub
